# One Size Does Not Fit All: Sociodemographic Factors Affecting Weight Loss in Adolescents

**DOI:** 10.1155/2020/3736504

**Published:** 2020-02-21

**Authors:** Claire B. Cummins, Kanika Bowen-Jallow, Sadia Tasnim, John Prochaska, Daniel Jupiter, Alex Wright, Byron D. Hughes, Omar Nunez-Lopez, Elizabeth Lyons, Andrea Glaser, Ravi S. Radhakrishnan, Debbe Thompson, Oscar E. Suman

**Affiliations:** ^1^Department of Surgery, University of Texas Medical Branch, Galveston, TX 77555, USA; ^2^School of Medicine, University of Texas Medical Branch, Galveston, TX 77555, USA; ^3^Department of Preventive Medicine and Community Health, University of Texas Medical Branch, Galveston, TX 77555, USA; ^4^Department of Nutrition and Metabolism, University of Texas Medical Branch, Galveston, TX 77555, USA; ^5^Department of Pediatrics, University of Texas Medical Branch, Galveston, TX 77555, USA; ^6^USDA/ARS Children's Nutrition Research Center, Department of Pediatrics, Baylor College of Medicine, Houston, TX 77030, USA

## Abstract

Successful lifestyle changes for weight reduction are heavily dependent on recognizing the importance of societal and cultural factors. Patients 13–19 years of age with a BMI ≥95^th^ percentile are eligible for our multidisciplinary adolescent weight loss clinic. A behavioral questionnaire was administered at the initial visit. Patients were seen every 4–6 weeks. Bivariate analysis was used to identify sociodemographic factors associated with differences in weight loss. Overall, receiving reduced cost meals was associated with a lower likelihood of losing weight (kg) (*p* < 0.01). When stratified by race, White adolescents were more likely to lose weight if caretakers reported having enough money to buy healthy food (*p* < 0.05); in contrast, Black adolescents were less likely to lose weight (*p* < 0.05). However, Black patients were more likely to lose weight if they reported eating fruits and vegetables (*p* < 0.05). Female adolescents were more likely to lose weight if they felt unhappy about their appearance (*p* < 0.05). Interestingly, male adolescents were less likely to lose weight if they felt unhappy about their appearance (*p* < 0.05). Social and cultural norms influence weight loss in adolescents in unique and differing ways. Culturally competent individualized interventions could increase weight loss in diverse groups of adolescents with obesity.

## 1. Introduction

Childhood obesity is a growing problem in the 21st century. According to the 2015-2016 National Health and Nutrition Examination Survey report, 18.5% of the youth of US aged 2 to 19 were obese [1]. A child or adolescent is considered obese when their BMI is at or above the 95^th^ percentile, with respect to sex-specific BMI-for-age growth charts provided by the Centers for Disease Control and Prevention (CDC) [2, 3]. A significant increase in obesity has been observed in youth over the last 20 years [1], and an important sequelae of childhood obesity is its propensity to result in adult obesity [4]. Obesity can lead to several severe comorbidities that were previously categorized as adult diseases including diabetes mellitus type 2 (DM2), cardiovascular disease (CVD), dyslipidemia, and nonalcoholic fatty liver disease/nonalcoholic steatohepatitis [5]. Among children with obesity, there is a threefold increase in hypertension (HTN) risk [6] and children with obesity that persists into adulthood have a five times higher risk of dying from CVDs [7]. In addition, the risk of adult cancers is increased by 9% per standard deviation increase in childhood BMI [4]. Adolescents that are overweight also have a higher risk of developing and an increased risk of mortality from colon cancer [8].

Apart from the significant comorbidities associated with childhood obesity, psychological, social, and behavioral consequences are also prevalent. Poor body image, low self-esteem, social isolation, discrimination, depression, and reduced quality of life are frequently observed in children with obesity [4]. There is an association between a range of psychosocial factors and increased risk of obesity, such as self-perception of being overweight and general unhappiness with their appearance [9–11]. Similarly, there have been a host of unhealthy weight control and dietary restrictions that have been reported to predict the development of obesity [10, 11]. Socioenvironmental factors also play a role, and parental obesity and unhealthy lifestyle are associated with adolescent obesity that persists into adulthood [12, 13].

Adolescent minorities in the US suffer obesity at a disproportionately higher rate than their Caucasian counterparts [14]. Nationwide, 15.7% of White, 18.8% of Black, and 21.6% of Hispanic children are obese [15]. This striking disparity is multifactorial and includes poor access to healthcare, cultural differences, socioeconomic status, and home environment [15–18]. Strategies to meet the needs of this at-risk group, therefore, require multifaceted interventions. Demographic factors affecting obesity and its treatment, such as socioeconomic status, race, and sex, have been extensively studied but there remains a paucity of evidence to suggest how sociodemographic factors might affect successful weight loss in adolescents [19].

Efforts to address adolescent obesity over the last decade have resulted in an increased number of multidisciplinary and multicomponent interventions [20–22]. Behavioral weight loss programs include nutrition and exercise recommendations in conjunction with behavioral therapy. Behavioral therapy aims to reduce maladaptive behaviors such as overeating and sedentary lifestyle and replace them with healthy behaviors such as portion control and exercise [23]. With promising results, family-based therapy has been recognized as the gold standard for treatment of adolescent obesity [23–25]. Program adherence by minorities and low-income populations presents a particular challenge due to cultural perspectives, community acceptance, childcare concerns, and financial constraints [26, 27].

The purpose of this study was to identify which sociodemographic and behavioral factors were associated with weight loss among a diverse group of adolescents. To our knowledge, this is the first study assessing these specific categories—effort, goal setting, technology utilization, and self-perception—in a multidisciplinary adolescent weight loss clinic.

## 2. Materials and Methods

### 2.1. Study Design

This was a post hoc analysis of data from the clinical registry of patients participating in the adolescent weight loss clinic at the University of Texas Medical Branch (UTMB Health). All patients were seen between 2016 and 2019. Patients were referred to the clinic by their primary care providers (PCPs) or other specialty providers and were instructed to follow-up every 4–6 weeks with the weight loss team composed of a pediatric surgeon, a pediatric gastroenterologist, a dietician, and a fitness instructor. Patients who had not presented within 10 weeks of their initial visit were considered lost to follow-up for visit 2, and patients who had not presented within 18 weeks of their initial visit were considered lost to follow-up for visit 3. Patients were screened for depression using the PHQ9 survey and referrals were made to a pediatric psychologist or psychiatrist when indicated. Anthropomorphic data was collected at each visit and laboratory data was collected when clinically indicated. Questionnaires were given separately to patients and caretakers on the first visit. The questionnaire was developed by faculty within the Departments of Preventive Medicine and Community Health, Nutrition and Metabolism, and Pediatrics and included past weight loss attempts, goal setting, dietary habits, activity level, family environment, and socioeconomic data. In the development of our questionnaire, several validated questionnaires were very influential, such as the body shape questionnaire (BSQ), body image assessment for obesity (BIA-O), multidimensional body-self relations questionnaire (MBSRQ), obesity related well-being (ORWELL-97), international physical activity questionnaire (IPAQ), and the diet satisfaction questionnaire (DSat-45). All data were deidentified and in accordance with our local IRB regulations for management of a clinical registry.

### 2.2. Study Cohort

All patients enrolled in the adolescent weight loss clinic were evaluated. Those eligible for evaluation in the adolescent weight loss clinic were patients aged 13–18 with sex-specific BMI-for-age ≥95^th^ percentile.

### 2.3. Outcomes

Primary outcomes measured were weight (kg) and BMI change (kg/m^2^). Anthropomorphic data and clinical data collected included height, weight, self-reported race, sex, age, BMI, systolic blood pressure (SBP), diastolic blood pressure (DBP), and presence of comorbidities. Comorbidities included HTN, gastroesophageal reflux disease (GERD), obstructive sleep apnea (OSA), DM2, and asthma. Height (cm) and weight (kg) were measured on the same scale and with the same stadiometer at each visit. Patients were measured in street clothes with no jackets, no shoes, and empty pockets.

### 2.4. Statistical Analysis

A paired *t*-test was used to evaluate weight and BMI changes from initial evaluation to the second and third visits. Data are presented as mean (standard deviation). Chi-squared analysis was used to evaluate demographic and questionnaire factors with follow-up, weight change, and BMI change. Data are presented as OR (95% CI). The goal of this study was not to generate a comprehensive model but to improve outcomes and attrition rates at our institution. In line with this goal, we sought to find specific questions that predicted weight and BMI loss in our diverse socioeconomic patient population.

## 3. Results and Discussion

### 3.1. Cohort Characteristics

A total of 189 children had an initial visit and were administered a survey questionnaire. Women and men were divided evenly at the initial visit, with 51% and 49%, respectively (Table 1). The plurality of patients was Hispanic (47%), followed by White (31%), Black (21%), and other races (1%). The average weight of all patients at visit 1 was 107.9 ± 25.9 kg with an average BMI of 39.2 ± 8.4 kg/m^2^ (Table 2).

A total of 109 patients had a second visit. The second visit occurred at a mean of 8.2 ± 5.3 weeks from the initial visit. Assuming a 10-week visit window from the initial visit, this represents a 68.9% retention rate. The average weight at visit 2 was 110.4 ± 27.5 kg with a BMI of 39.5 ± 8.6 kg/m^2^. The majority of patients at visit 2 lost weight (kg) (69%) and had a reduced BMI (74%). The average weight change at visit 2 was -0.91 ± 2.7 kg (*p* < 0.001) with an average BMI change of −0.49 ± 0.91 kg/m^2^ (*p* < 0.001). There was no significant difference in the demographic composition of patients at visit 2 from baseline.

A total of 68 patients had a third visit. The third visit occurred at a mean of 15.5 ± 7.2 weeks from the initial visit. Assuming an 18-week visit window from the initial visit, this represents a 51.5% retention rate. The average weight at visit 3 was 107.9 ± 29.8 kg with a BMI of 38.9 ± 9.4 kg/m^2^. The majority of patients at visit 3 lost weight (kg) (62%) and had a reduced BMI (75%). The average weight change at visit 3 was −1.23 ± 4.1 kg (*p*=0.02) with an average BMI change of −0.81 ± 1.4 kg/m^2^ (*p* < 0.001). There was no significant difference in the demographic composition of patients at visit 3 from baseline.

Comorbidities in the patient population were rare (Table 3). Asthma was the most common comorbid condition (14%), followed by OSA (13%), HTN (5%), DM2 (5%), and GERD (2%). When stratified by race and sex, no significant differences between the demographic groups were seen.

### 3.2. Racial, Ethnic, and Sex Differences in Survey Responses

**Figure 1:**
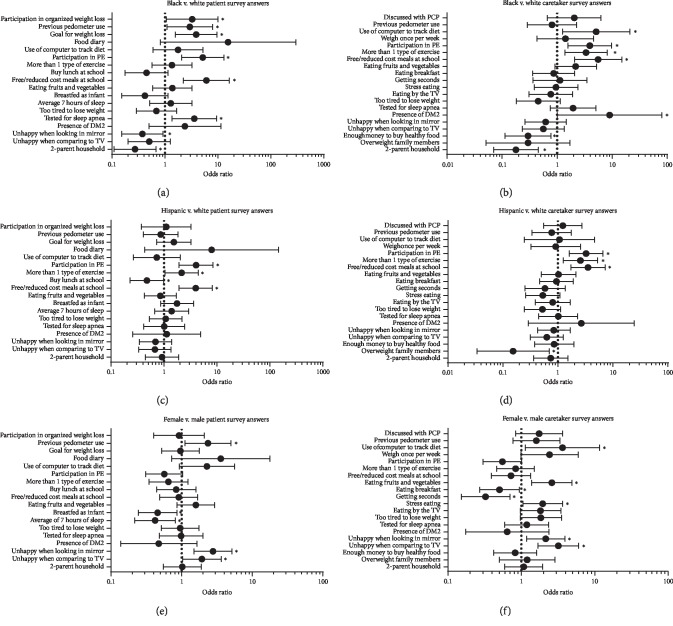
Racial, ethnic, and sex differences in survey responses. Results presented as odds ratio ±95% confidence interval. PE—physical education, DM2—diabetes mellitus type 2, TV—television, and PCP—primary care provider.

When examining survey responses, statistically significant differences were noted between races and different ethnicities. Black patients were significantly more likely to report receiving free or reduced cost meals at school (*p* < 0.001), less likely to report feeling unhappy when looking in the mirror (*p*=0.03), and less likely to have a two-parent household (*p*=0.008) when compared to White patients (Figure 1(a)). Compared to caretakers of White patients, caretakers of Black patients were more likely to report receiving free or reduced cost meals at school (*p* < 0.001) and less likely to report having enough money to purchase healthy food (*p*=0.02) (Figure 1(b)). Other significant results are presented in Figures 1(a) and 1(b).

Hispanic patients were significantly more likely to report receiving free or reduced cost meals at school (*p* < 0.001) than White patients (Figure 1(c)). However, Hispanic patients were less likely to report buying lunch at school (*p*=0.04) than White patients. Caretakers of Hispanic patients were more likely to report that patients received free or reduced cost meals at school (*p* < 0.001) than caretakers of White patients (Figure 1(d)). Other significant results are presented in Figures 1(c) and 1(d).

Female patients were significantly more likely to report feeling unhappy when looking in the mirror (*p*=0.002) and feeling unhappy when comparing their looks to those on TV or in magazines (*p*=0.04) than male patients (Figure 1(e)). Caretakers of female patients were more likely to report a patient feeling unhappy when looking in the mirror (*p*=0.02) and a patient feeling unhappy when comparing looks to those on TV or in magazines (*p* < 0.001) than caretakers of male patients (Figure 1(f)). Other significant results are presented in Figures 1(a) and 1(b).

### 3.3. Survey Responses Predictive of Weight and BMI Reduction

**Figure 2:**
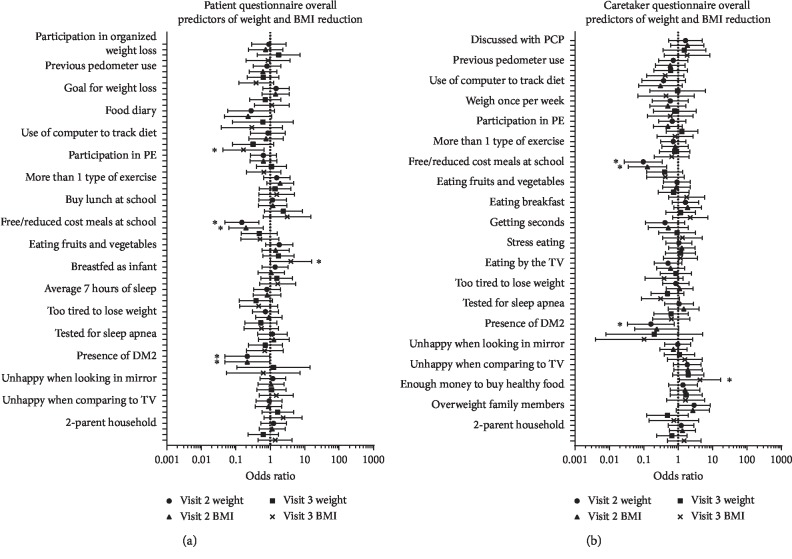
Survey responses predictive of weight and BMI reduction. Results presented as odds ratio ±95% confidence interval. PE—physical exercise, DM2—diabetes mellitus type 2, TV—television, and PCP—primary care provider.

Weight loss (kg) was significantly less likely if patients or caretakers reported receiving free or reduced cost meals at school (*p* < 0.001 and *p* < 0.001, patients and caretakers, respectively) (Figures 2(a) and 2(b)). Reduced BMI was significantly less likely if patients or caretakers reported receiving free or reduced cost meals at school (*p*=0.003 and *p* < 0.001, patients and caretakers, respectively) (Figures 2(a) and 2(b)). Reduced BMI was significantly more likely if patients reported eating 2-3 servings of fruit and vegetables per day (*p* < 0.05) or caretakers reported that patients felt unhappy when comparing their looks to those on TV or in magazines (*p*=0.04) (Figures 2(a) and 2(b)). Other significant results are presented in Figures 2(a) and 2(b).

### 3.4. Racial and Ethnic Survey Responses Predictive of Weight and BMI Reduction

**Figure 3:**
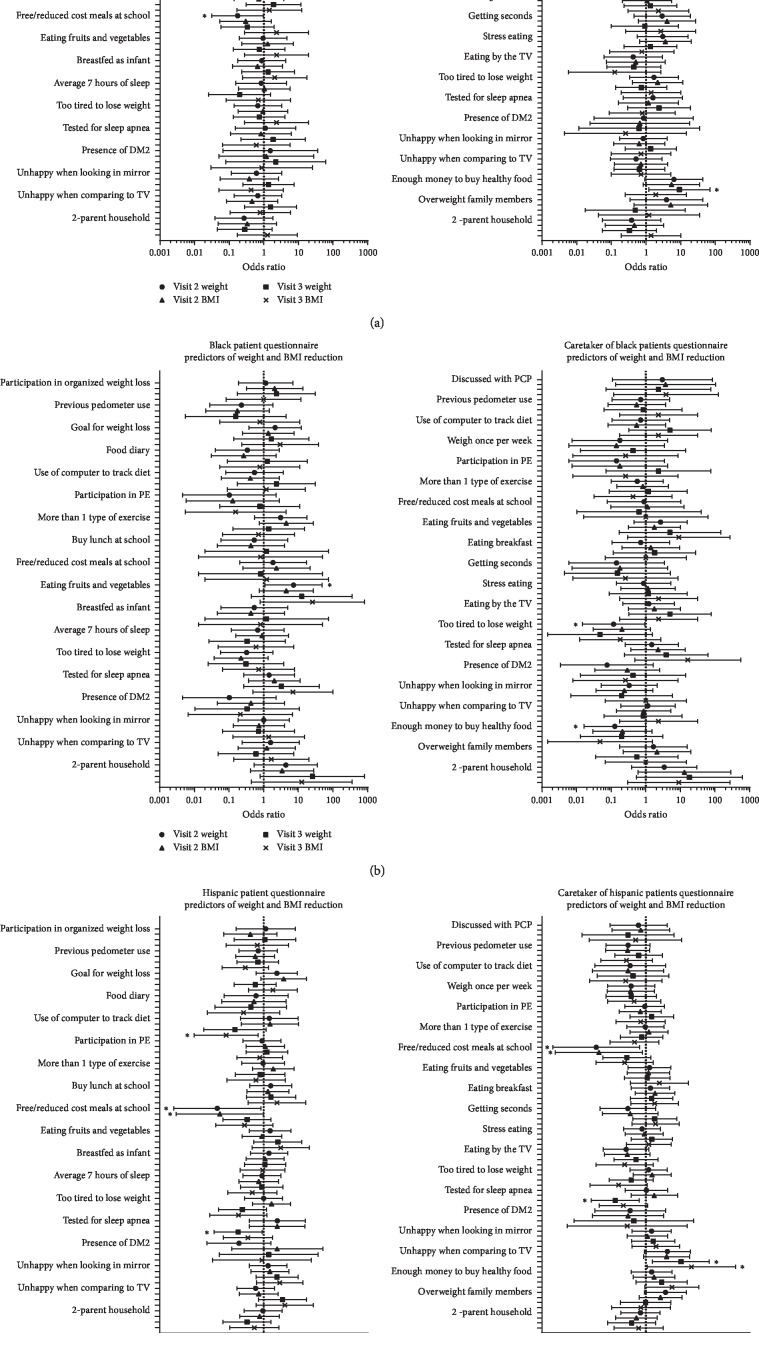
Racial and ethnic survey responses predictive of weight and BMI reduction. Results presented as odds ratio ±95% confidence interval. PE—physical exercise, DM2—diabetes mellitus type 2, TV—television, and PCP—primary care provider.

When stratified by race, additional predictors were found. White patients were significantly less likely to lose weight (kg) if they received free or reduced cost meals at school (*p*=0.04) or if caretakers reported having enough money to buy healthy food (*p*=0.03) (Figure 3(a)). Additional significant results are presented in Figure 3(a).

Black patients were more likely to lose weight (kg) if patients reported eating 2-3 servings of fruits or vegetables per day (*p*=0.03) (Figure 3(b)). Conversely, Black patients were less likely to lose weight at visit 2 if caretakers reported patients being too tired to lose weight (*p* < 0.05). In contrast to the findings among White patients, Black patients were significantly less likely to lose weight if caretakers reported that they had enough money to buy healthy food (*p*=0.04).

Hispanic patients were less likely to lose weight (kg) if patients or caretakers reported that patients had been tested for obstructive sleep apnea (*p*=0.04 and *p*=0.01, patients and caretakers, respectively) (Figure 3(c)). Hispanic patients were more likely to lose weight at visit 3 if caretakers reported that patients were unhappy with their looks when comparing themselves to what they saw on TV or in magazines (*p*=0.02). Hispanic patients were also less likely to lose weight if patients or caretakers reported receiving free or reduced cost meals at school (*p* < 0.05 and *p* < 0.05, patients and caretakers, respectively). Additional significant findings are presented in Figure 3(c).

### 3.5. Sex Survey Responses Predictive of Weight and BMI Reduction

**Figure 4:**
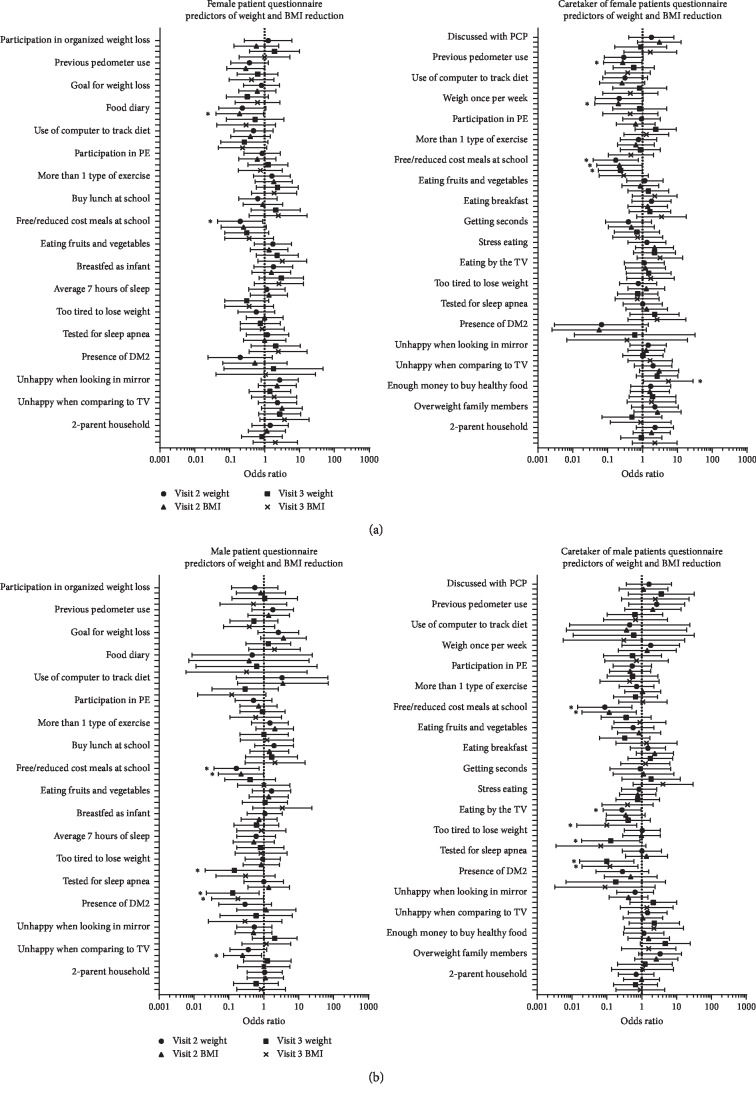
Sex survey responses predictive of weight and BMI reduction. Results presented as odds ratio ±95% confidence interval. PE—physical exercise, DM2—diabetes mellitus type 2, TV—television, and PCP—primary care provider.

Female patients were less likely to have lost weight if patients or caretakers reported receiving free or reduce d cost meals at school (*p*=0.03 and *p*=0.02) (Figure 4(a)). Reduced BMI was significantly less likely if caretakers reported receiving free or reduced cost meals at school (*p*=0.04). Female patients were more likely to lose weight if caretakers reported that they were unhappy when comparing their looks to what they saw on TV or in magazines (*p*=0.04). Other significant findings are presented in Figure 4(a).

Male patients were less likely to lose weight if patients or caretakers reported receiving free or reduced cost meals at school (*p*=0.02 and *p*=0.01, patients and caretaker, respectively) (Figure 4(b)). Male patients were less likely to have a reduced BMI if patients or caretakers reported receiving free or reduced cost meals at school (*p*=0.04 and *p*=0.02, patients and caretakers, respectively) or if patients reported being unhappy when they looked in the mirror (*p*=0.03). Male patients were significantly less likely to lose weight if patients or caretakers reported that patients were too tired to lose weight (*p* < 0.05 and *p*=0.04, patients and caretakers, respectively) or if patients or caretakers reported that the patient had been tested for sleep apnea (*p*=0.02 and *p*=0.01, patients and caretakers, respectively). Male patients were significantly less likely to have a reduced BMI if patients or caretakers reported that the patient had been tested for sleep apnea (*p* < 0.05 and *p*=0.02, patients and caretakers, respectively). Additional significant findings are presented in Figure 4(b).

## 4. Conclusions

Our study successfully identified several statistically significant predictors of weight loss in adolescents. As expected, responses to our initial visit questionnaire were significantly different between demographic groups, indicating that the barriers to weight loss are varied and unique to each group. In addition, these results highlight areas for improvement in tailoring behavioral, dietary, and activity changes to specific demographic groups of adolescents. Example areas for improvement include reformation of the National Student Lunch Program (NSLP), emphasizing healthy body images for male adolescents, providing culturally competent healthy recipes for minority families, and an increased emphasis on proper sleep hygiene. Critically examining the results of our clinic after implementing these changes will provide an opportunity for improvement on a continuous basis.

Receiving free or reduced cost school lunches was associated with significantly lower odds of weight and BMI reduction. This association was present in both males and females. Racial and ethnic stratification showed that Hispanic and Black patients were significantly more likely to report receiving free or reduced cost school lunches than White patients; but receiving free or reduced cost school lunches was only significantly associated with reduced odds of weight and BMI reduction in White and Hispanic patients. Notably, surrogate indicators of socioeconomic status, such as family income, amount spent on groceries weekly, and amount spent on restaurants weekly, were not found to be significant predictors of weight or BMI reduction. The NSLP has long been known to have no protective effects for childhood obesity, even after addressing the selection bias caused by differences in NSLP participants and nonparticipants [24]. This is likely due to the fact that less than one-third of schools participating in the NSLP met the standards for calories obtained from fat or saturated fat in the average lunch [25]. NSLP participants also consumed significantly more calories per day than nonparticipants and had significantly increased sodium intake [28]. In states with laws that encourage or require more stringent nutritional policies than the federal policy dictates, students who obtained school lunches, particularly those eligible for a free or reduced cost lunch, had a more favorable weight status [29]. Other countries may also serve as a model for further development of the NSLP as well. In Japan, children are more likely to obtain an appropriate level of nutritional intake when lunch is provided than when it is not; school lunch programs help to increase weight in underweight children and decrease weight in overweight children [30, 31]. Reforms to the NSLP program could represent a useful intervention to prevent and treat childhood obesity. However, other methods will still be necessary since overall energy intake can easily be influenced by other meals and snacks.

Females were significantly more likely than males to report feeling unhappy when looking in the mirror or comparing their looks to those they see on television or in magazines. When stratified by sex, these feelings were associated with a significantly higher likelihood of having a reduced BMI. In males, however, feeling unhappy with their looks was associated with a significantly reduced likelihood of reduced BMI. This evidence suggests that body image issues may represent a larger roadblock to weight loss in adolescent males than females. Body image issues may also be underrecognized in the adolescent male community. In adults, men are as likely as women to seek care for body dysmorphic disorder, but they are less likely to receive treatment [32]. One study found a slightly higher prevalence of body dysmorphic disorder in male students [33]. Additionally, areas of concern for adult and adolescent males are most frequently centered on being too small or unmuscular, while females are most frequently centered around being overweight [32–34]. Increased education and emphasis on a healthy body image for males could help improve weight loss outcomes. It is also possible that there is a disparity between the definition of happy between male and female adolescents with obesity. Exploring whether this distinction exists would be a great topic for future studies.

Caretaker-reported ability to afford healthy food was a significant predictor of weight loss among White patients; however, in Black patients, it was associated with a significantly reduced likelihood of weight loss. Interestingly, if Black patients reported eating several servings of fruits and vegetables per day, they had increased odds of weight loss. The ability to afford healthy food in our population may not be a deciding factor for weight loss in Black patients, and weight loss may instead be dependent on cultural factors in the Black American population. Studies have demonstrated that, in general, Black Americans are more accepting of larger body sizes [35–37]. Dietary acculturation is the extent to which minority groups adopt the dietary customs of the majority culture [38]. Several studies have shown that diet composition, including the consumption of fruits and vegetables, can be heavily influenced by dietary acculturation [39, 40]. Increased emphasis with Black adolescents on incorporating fruits and vegetables into current dietary practices could be especially beneficial for increasing weight loss in this demographic. However, the process of acculturation is often inconsistent and does not always fit an expected pattern [37]. Care must then be taken to ensure that dietary acculturation is a net positive influence on the health of Black Americans. Studies in other disease processes have shown that simple increase in consumption of fruits and vegetable may help with weight loss due to exclusion of other food groups [41, 42].

Black patients and Hispanic patients were less likely to lose weight if caretakers reported that they had been tested for OSA. Similarly, male patients were less likely to lose weight or have a reduced BMI if patients or caretakers reported testing for OSA. Black patients and male patients were also less likely to lose weight if they reported being too tired for weight loss. These survey questions are interlocked since patients with obesity who reported being tired are those who are most likely referred for sleep apnea testing [43]. These findings are interesting since they may contradict the literature on the outcomes of treatment for obstructive sleep apnea in adults. Daily physical activity and body weight in adults were not affected after successful treatment of OSA with continuous positive airway pressure (CPAP), even though quality of life and depressive symptoms were alleviated [44, 45]. One study even showed that total energy expenditure went down after successful treatment of OSA with CPAP due to the decreased energy expenditure at night from less strenuous breathing patterns [44]. It is possible that feeling too tired to lose weight is a psychological barrier to weight loss in these patients. Increased proper sleep hygiene may improve weight loss in Black patients, Hispanic patients, and male patients.

This study had several limitations. The attrition rate for our adolescent weight loss clinic, although similar to that reported in other weight loss clinics [46], remains high. Improving our cultural competence could help decrease attrition rates and was a major impetus in conducting this analysis as a quality improvement project. Our clinic patients demonstrated a significant reduction in weight over the course of the examined period. While this indicates clinical success, this may have skewed our results towards adolescents who successfully lost weight. Halting weight gain, in addition to naturally occurring height growth in the adolescent population, can be just as important as weight loss. As a single institution, our results may not be generalizable to the rest of the nation. However, the demographics of our adolescent weight loss clinic mirror that of the adolescent population with obesity of the United States [1]. Similarly, since all patients were a part of our multidisciplinary weight loss clinic, these results may not be generalizable to treatment and counseling of adolescents with obesity in other settings. Development of a predictive model would improve generalizability of our study and will be the focus of future research. Our weight loss clinic EMR had the additional limitation of being unable to separate race and ethnicity. More nuanced results could be obtained if race and ethnicity were analyzed separately. Finally, there was occasionally a disparity between weight and BMI reduction in our patients. Since adolescents have not yet reached full height, a stable BMI or a small reduction in BMI have been associated with an improvement in cardiovascular risk factors, DM2, and liver disease [47]. BMI may be a more sensitive tool for gauging obesity improvement in adolescents, but since it relies on two measurements, height and weight, it may not be as reliably accurate as weight alone in a clinic setting and the smaller effect size may result in additional power being necessary to detect statistically significant changes, especially over a shorter time period.

## Figures and Tables

**Table 1:** Cohort demographic composition.

Demographic	Visit 1	Visit 2	Visit 2	Visit 3	Visit 3
	*N* (%)	*N* (%)	OR (95% CI)	*N* (%)	OR (95% CI)
Total	189	109		68	
Sex					
Female (ref.)	96 (51%)	55 (50%)		37 (54%)	
Male	93 (49%)	54 (50%)	1.22 (0.70–2.13)	31 (46%)	1.25 (0.9–2.28)
Race					
White (ref.)	58 (31%)	37 (34%)		21 (31%)	
Black	40 (21%)	20 (18%)	0.57 (0.25–1.29)	9 (13%)	0.51 (0.20–1.28)
Hispanic	89 (47%)	51 (47%)	0.76 (0.39–1.50)	38 (56%)	1.31 (0.66–2.59)
Weight loss (kg)^*∗*^		75 (69%)		42 (62%)	
Reduced BMI (kg/m^2^)^#^		81 (74%)		51 (75%)	

^*∗*^Proportion of patients who achieved weight loss at visit 2 and visit 3, respectively. ^#^Proportion of patients who achieved weight loss at visit 2 and visit 3, respectively.

**Table 2:** Cohort anthropomorphic composition.

	Initial visit	Visit 2^*∗*^	Visit 3^*∗*^
Weight (kg ± SD)	107.9 ± 25.9	110.4 ± 27.5	107.9 ± 29.8
Weight change (kg ± SD)		−0.91 ± 2.7	−1.23 ± 4.1
BMI (kg/m^2^ ± SD)	39.2 ± 8.4	39.5 ± 8.6	38.9 ± 9.4
BMI change (kg/m^2^ ± SD)		−0.49 ± 0.91	−0.81 ± 1.4
Time from initial visit (weeks ± SD)		8.2 ± 5.3	15.5 ± 7.2

^*∗*^Data representative of patients who have completed the stated number of visits. Change statistics were generated with paired *t*-test analysis comparing patients to their initial visits.

**Table 3:** Presence of comorbidities.

	*N* (%)	OR (95% CI)
HTN	9 (5%)	
White (ref.)	4 (8%)	
Black	2 (6%)	0.82 (0.14–4.7)
Hispanic	3 (4%)	0.47 (0.10–2.2)
Female (ref.)	3 (4%)	
Male	6 (7%)	2.1 (0.51–8.8)
OSA		
White (ref.)	9 (17%)	
Black	10 (31%)	2.22 (0.79–6.3)
Hispanic	6 (8%)	0.40 (0.13–1.2)
Female (ref.)	13 (15%)	
Male	12 (15%)	1.1 (0.45–2.5)
Asthma		
White (ref.)	8 (15%)	
Black	9 (28%)	2.2 (0.75–6.5)
Hispanic	9 (11%)	0.70 (0.25–2.0)
Female (ref.)	13 (15%)	
Male	13 (16%)	1.1 (0.45–2.47)
GERD		
White (ref.)	1 (2%)	
Black	0 (0%)	0.54 (0.021–13.6)
Hispanic	3 (4%)	2.0 (0.20–19.8)
Female	2 (2%)	
Male	2 (2%)	0.98 (0.13–7.1)
DM2		
White (ref.)	2 (4%)	
Black	4 (13%)	3.64 (0.63–21.2)
Hispanic	3 (4%)	0.98 (0.16–6.1)
Female (ref.)	4 (5%)	
Male	5 (6%)	0.77 (0.20–3.0)

HTN—hypertension, OSA—obstructive sleep apnea, GERD—gastroesophageal reflux disease, and DM2—diabetes mellitus type 2.

## Data Availability

The deidentified survey and clinical data used to support the findings of this study are available from the corresponding author upon request.
